# RETRACTED: Yang et al. How Does Physical Activity Enhance the Subjective Well-Being of University Students? A Chain Mediation of Cognitive Reappraisal and Resilience. *Behav. Sci.* 2024, *14*, 164

**DOI:** 10.3390/bs14090753

**Published:** 2024-08-27

**Authors:** Mengmeng Yang, Ji Wu, Yigang Wu, Xinxing Li

**Affiliations:** 1 School of Physical Education, Shanghai University of Sport, Shanghai 200438, China; 2 School of Economics and Management, Shanghai University of Sport, Shanghai 200438, China; 3 Health and Exercise Science Laboratory, Department of Physical Education, Seoul National University, Seoul 08826, Republic of Korea

The journal *Behavioral Sciences* retracts the article titled, “How Does Physical Activity Enhance the Subjective Well-Being of University Students? A Chain Mediation of Cognitive Reappraisal and Resilience” [1], cited above.

Following publication, concerns were brought to the attention of the Editorial Office regarding an unattributed overlap between this publication [1] and an earlier article [2] published by a different group of authors in another language.

Adhering to our complaints procedure, an investigation was conducted by the Editorial Office and Editorial Board which confirmed a significant overlap between these two publications [1,2], without the appropriate acknowledgement or citation. The Editorial Board has decided to retract this article as per MDPI’s retraction policy (https://www.mdpi.com/ethics#_bookmark30) (accessed on: 12 March 2024) and in line with the committee on Publication Ethics’ retraction guideline (https://publicationethics.org/retraction-guidelines) (accessed on: 12 March 2024).

This retraction was approved by the Editor-in-Chief of the journal *Behavioral Sciences.*

The authors have agreed to this retraction. 
